# Bowel necrosis following endovascular revascularization for chronic mesenteric ischemia: a case report and review of the literature

**DOI:** 10.1186/1471-230X-13-118

**Published:** 2013-07-19

**Authors:** Takuro Shirasu, Akihiro Hosaka, Hiroyuki Okamoto, Kunihiro Shigematsu, Yasushi Takeda, Tetsuro Miyata, Toshiaki Watanabe

**Affiliations:** 1 Division of Vascular Surgery, Department of Surgery, Graduate School of Medicine, The University of Tokyo, Tokyo, Japan; 2 Department of Surgery, Tokyo Rosai Hospital, Tokyo, Japan; 3 Department of Surgical Oncology, The University of Tokyo, Tokyo, Japan

**Keywords:** Chronic mesenteric ischemia, Endovascular revascularization, Embolization, Bowel necrosis, Embolic protection device

## Abstract

**Background:**

Endovascular revascularization has recently been established as a less invasive treatment method for chronic mesenteric ischemia. However, intestinal necrosis caused by distal embolization following this procedure has not been emphasized.

**Case presentation:**

The present report describes a 59-year-old man who was treated with endovascular revascularization for chronic mesenteric ischemia. After the procedure, he was diagnosed with intestinal necrosis caused by distal embolization. Despite emergent bowel resection, he died on postoperative day 109.

**Conclusion:**

Although endovascular revascularization for chronic mesenteric ischemia is less invasive and may be suitable for high-risk patients, attention should be paid to avoid embolic complications that can cause intestinal infarction possibly leading to a fatal condition.

## Background

Endovascular revascularization (ER) is an emerging treatment alternative for chronic mesenteric ischemia (CMI) [1]. Although open surgical revascularization (OR) yields a satisfactory outcome with respect to symptom relief and primary patency, it can be associated with perioperative morbidity and mortality. ER is advantageous in that it is less invasive [2,3]. However, procedure-related complications following ER occur in approximately 10% of cases. Of these complications, distal embolization and subsequent bowel necrosis can lead to a fatal condition [4-8]. The present report describes a case of CMI associated with intestinal infarction after ER and discusses the procedure-specific complications.

## Case presentation

**Figure 1:**
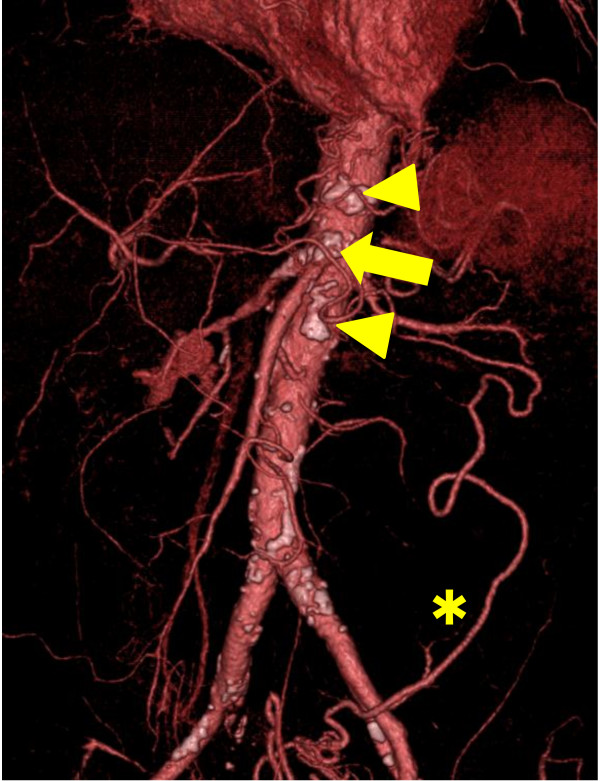
**Computed tomography before the intervention.** The arrow indicates occlusion of the superior mesenteric artery. The arrowheads indicate obstruction of the celiac and inferior mesenteric arteries. The asterisk shows the development of a collateral vessel from the left iliac artery.

A 59-year-old man with postprandial abdominal pain, diarrhoea, and vomiting was referred to our hospital. His body mass index was 18.4. His symptoms improved with total parenteral nutrition but relapsed after he resumed his normal diet. He had a history of multiple abdominal surgical interventions. During the past 20 years, he had undergone open drainage for acute pancreatitis, cholecystectomy and choledochotomy for acute cholangitis, cystogastrostomy for pancreatic pseudocyst, and choledocho-jejunostomy for bile duct stenosis. He had a history of hypertension, diabetes, and cervical spondylosis, as well as a history of smoking. An upper gastrointestinal endoscopy showed no ischemic change, whereas total colonoscopy revealed ischemia in the distal ileum and the ascending colon. Enhanced abdominal computed tomography (CT) showed stenosis of the superior mesenteric artery (SMA) and occlusion of the celiac and inferior mesenteric arteries with developed collateral vessels from the SMA and left iliac artery (Figure 1).

**Figure 2:**
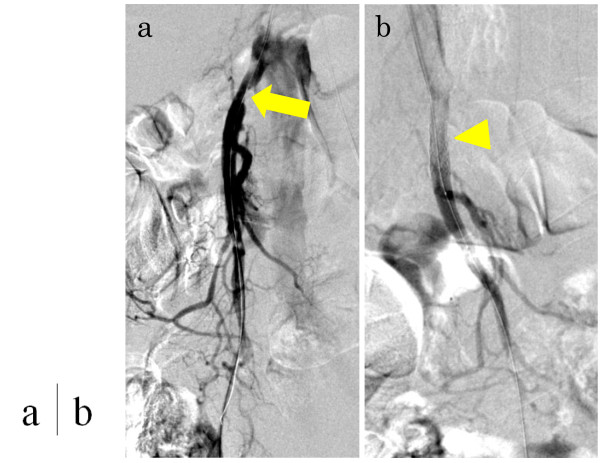
**Digital subtraction angiography during percutaneous transluminal angioplasty and stenting. (a)** Stenosis was confirmed near the root of the superior mesenteric artery (arrow). **(b)** After dilatation, a stent (6 mm × 16 mm) was inserted (arrowhead).

The patient was diagnosed with CMI caused by splanchnic arterial stenoses and occlusions, and revascularization of the SMA was necessary. We chose ER for this treatment, taking into consideration his poor nutritional condition and hostile abdomen. The root of the SMA was highly calcified; therefore, we considered angioplasty with stenting to be appropriate. The procedure was performed under local anaesthesia via the left brachial route. The stenosis close to the origin of the SMA was traversed with a 0.014-inch guidewire. After systemic administration of 3000 units of unfractionated heparin, the lesion was predilated, and a stent (Palmaz Genesis, 6 mm × 16 mm, Cordis/Johnson & Johnson, Miami, FL, USA) was placed without apparent difficulty (Figure 2). He subsequently received a continuous infusion of 15000 units of unfractionated heparin for the first 24 hours and 100 mg of aspirin per day.

The patient complained of right upper quadrant abdominal pain after the intervention, and his white blood cell count was elevated to 20 200/μL on the next day. Muscular guarding was present with further elevation of white blood cell count on the second day, and CT revealed small bowel necrosis with intestinal pneumatosis and bloody ascites. Emergency laparotomy was performed, and the necrotic ileum and ischemic ascending colon were totally resected. Post operatively, the patient suffered an ischemic heart attack and liver abscess, which were both treated nonsurgically. Although an ileostomy and a mucous fistula of the colon were viable without ischemia throughout the postoperative course, bacterial translocation from the intestine, which had already been damaged by chronic ischemia before ER, was suspected as a cause of the liver abscess. He died of massive gastrointestinal bleeding on postoperative day 109.

### Discussion

Abdominal angina or CMI is characterized by postprandial abdominal pain and weight loss. Insufficient intestinal blood flow causes these symptoms and is usually produced by the obstruction of 2 or 3 splanchnic vessels [7]. The underlying etiology of this disease is atherosclerosis in more than 90% of cases. Other causes include fibromuscular dysplasia, vasculitis (such as Takayasu arteritis, giant cell arteritis, polyarteritis nodosa, systemic lupus erythematosus, and thromboangiitis obliterans), malignancy, and radiation. Patients are initially treated with conservative therapy including bowel resting, smoking cessation, and administration of vasodilator drugs. Revascularization is considered if these conservative treatments fail to relieve the symptoms. Open surgery has been the standard method for revascularization in CMI. On the other hand, since the first report by Furrer et al. on the effectiveness of percutaneous transluminal angioplasty for CMI [2], ER has been considered as the treatment of choice in some cases [9]. Previous studies have reported the equivalent technical success and symptom relief rates, and lower morbidity in ER, compared with OR. Additionally, the primary patency rate is lower in ER than in OR, resulting in more secondary interventions [2,3]. Therefore, ER is currently recommended for high-risk patients [8]. The therapeutic method for CMI should be carefully determined, because the treatment outcomes are often affected by systemic cardiovascular comorbidities, as in our patient.

Patients treated with ER can have associated procedure-specific complications, and sometimes follow catastrophic courses, as in the present case. Distal embolization and arterial dissection are often critical conditions. Eight cases of distal embolization as a complication of ER for CMI have been previously reported [4-8]. All of these patients underwent bowel resection, and 7 of 8 patients died of postoperative multiple organ failure (Table 1). Bowel necrosis and subsequent sepsis can easily deteriorate the general condition of patients considered high risk for OR, causing 60% of all deaths after ER for CMI [4-8]. Therefore, efforts should be made to prevent embolization.

**Table 1:** Review of distal embolization after endovascular revascularization for chronic mesenteric ischemia

**Author, year**	**Patients included (n)**	**Branches treated (n)**	**Patients with morbidity (n)**	**Embolization (n)**	**Bowel necrosis (n)**	**Death caused by embolization (n)**	**Patients with mortality (n)**
Sarac, 2008 [4]	65	87	20	3	3	3	5
Zerbib, 2008 [5]	14	31	3	1	1	1	2
Kasirajan, 2001 [6]	28	32	5	2	2	2	3
Allen, 1996 [7]	19	24	1	1	1	1	1
Rose, 1995 [8]	8	9	2	1	1	0	1

Little is known about the impact of stent placement in terms of distal embolization. We did not use an embolic protection device (EPD), because there is little evidence showing its efficacy during ER for CMI. An EPD may be useful in some cases with CMI [10,11]. Brown et al. [11] reported the feasibility of routine EPD use because there was no major perioperative morbidity in their endovascular treatment. In percutaneous revascularization and stent placement for renal artery stenosis, several reports have suggested the efficacy of EPDs [12,13]. These studies showed that embolic particles were observed in 60–90% of cases treated for atherosclerotic renal arteries. However, EPD use involves the intrinsic complications of vasospasm, arterial dissection, arterial wall damage, distal hypoperfusion, and even distal embolization [14,15]. Further studies are necessary to verify the usefulness of EPDs in cases with CMI.

## Conclusion

Although ER for CMI is less invasive and may be suitable for high-risk patients, attention should be paid to avoid embolic complications.

## Consent

Written informed consent was obtained from the patient and his family for publication of this case report and any accompanying images. A copy of the written consent is available for review by the Editor-in-Chief of this journal.

## Abbreviations

CMI: Chronic mesenteric ischemia; CT: Computed tomography; ER: Endovascular revascularization; OR: Open surgical revascularization; SMA: Superior mesenteric artery; EPD: Embolic protection device

## Competing interests

We have no conflict of interest to declare.

## Authors’ contributions

TS collected data and wrote the paper. HO, KS, YT and TW made substantial contributions to patient management and supervised the manuscript. AH and TM critically revised the article. All authors read and approved the manuscript.

## Pre-publication history

The pre-publication history for this paper can be accessed here:

http://www.biomedcentral.com/1471-230X/13/118/prepub

## References

[B1] Biolato M Miele L Gasbarrini G Grieco A Abdominal angina Am J Med Sci 2009 338 389 395 10.1097/MAJ.0b013e3181a85c3b 19794303

[B2] Furrer J Grüntzig A Kugelmeier J Goebel N Treatment of abdominal angina with percutaneous dilatation of an arteria mesenterica superior stenosis. Preliminary communication Cardiovasc Intervent Radiol 1980 3 43 44 10.1007/BF02551961 7371046

[B3] van Petersen AS Kolkman JJ Beuk RJ Huisman AB Doelman CJ Geelkerken RH Multidisciplinary Study Group of Splanchnic Ischemia. Open or percutaneous revascularization for chronic splanchnic syndrome J VascSurg 2010 51 1309 1316 10.1016/j.jvs.2009.12.064 20304586

[B4] Sarac TP Altinel O Kashyap V Bena J Lyden S Sruvastava S Eagleton M Clair D Endovascular treatment of stenotic and occluded visceral arteries for chronic mesenteric ischemia J Vasc Surg 2008 47 485 491 10.1016/j.jvs.2007.11.046 18295100

[B5] Zerbib P Lebuffe G Sergent-Baudson G Chamatan A Massouille D Lions C Chambon JP Endovascular versus open revascularization for chronic mesenteric ischemia: a comparative study Langenbecks Arch Surg 2008 393 865 870 10.1007/s00423-008-0355-x 18575885

[B6] Kasirajan K O’Hara PJ Gray BH Hertzer NR Clair DG Greenberg RK Leonard P Krajewski LP Beven EG Ouriel K Chronic mesenteric ischemia: open surgery versus percutaneous angioplasty and stenting J Vasc Surg 2001 33 63 71 10.1067/mva.2001.111808 11137925

[B7] Allen RC Martin GH Rees CR Rivera FJ Talkington CM Garrett WV Smith BL Pearl GJ Diamond NG Lee SP Thompson JE Mesenteric angioplasty in the treatment of chronic intestinal ischemia J Vasc Surg 1996 24 415 421 10.1016/S0741-5214(96)70197-0 8808963

[B8] Rose SC Quigley TM Raker EJ Revascularization for chronic mesenteric ischemia: comparison of operative arterial bypass grafting and percutaneous transluminal angioplasty J Vasc Interv Radiol 1995 6 339 349 10.1016/S1051-0443(95)72819-6 7647433

[B9] American Gastroenterological Association Medical Position Statement Guidelines on intestinal ischemia Gastroenterology 2000 118 951 953 10784595 10.1016/s0016-5085(00)70182-x

[B10] Oderich GS Malgor RD Ricotta JJ 2nd Open and endovascular revascularization for chronic mesenteric ischemia: tabular review of the literature Ann Vasc Surg 2009 23 700 712 10.1016/j.avsg.2009.03.002 19541451

[B11] Brown DJ Schermerhorn ML Powell RJ Fillinger MF Rzucidlo EM Walsh DB Wyers MC Zwolak RM Cronenwett JL Mesenteric stenting for chronic mesenteric ischemia J VascSurg 2005 42 268 274 10.1016/j.jvs.2005.03.054 16102625

[B12] Thatipelli MR Misra S Sanikommu SR Schainfeld RM Sharma SK Soukas PA Embolic protection device use in renal artery stent placement J Vasc Interv Radiol 2009 20 580 586 10.1016/j.jvir.2009.01.025 19328725 PMC3752422

[B13] Holden A Hill A Jaff MR Pilmore H Renal artery stent revascularization with embolic protection in patients with ischemic nephropathy Kidney Int 2006 70 948 955 10.1038/sj.ki.5001671 16837918

[B14] Bates MC Campbell JE Pitfalls of embolic protection Tech Vasc Interv Radiol 2011 14 101 107 10.1053/j.tvir.2011.01.008 21550513

[B15] Müller-Hülsbeck S Schäfer PJ Hümme TH Charalambous N Elhöft H Heller M Jahnke T Embolic protection devices for peripheral application: wasteful or useful? J EndovascTher 2009 16 Suppl 1:I 163 169 10.1583/08-2596.1 19317576

